# Promoting adoption of two-stage agricultural drainage ditches: A change agent perspective

**DOI:** 10.1371/journal.pone.0229969

**Published:** 2020-03-09

**Authors:** Pranay Ranjan, Jonathan D. Witter

**Affiliations:** 1 Department of Forestry & Natural Resources, Purdue University, West Lafayette, Indiana, United States of America; 2 Ohio State Agricultural Technical Institute, Wooster, Ohio, United States of America; Universidad de Murcia, SPAIN

## Abstract

Adoption of innovations, including adoption of conservation practices, is a topic of extensive scholarly enterprise. The diffusion of innovations literature has often examined the characteristics of three sets of variables: the adopter, the change agent, and the innovation. This literature clearly establishes the crucial role of change agents in promoting an innovation. However, what we don’t know is what makes change agents want to promote a particular innovation. In this study, change agents’ perceptions of the attributes of two-stage drainage ditches, an innovative agricultural drainage ditch design, are examined in order to understand what affects their willingness to promote them. Diffusion of innovation theory provides the conceptual grounding as well as the theoretical motivation for this study. The data for this study come from semi-structured interviews with 17 change agents. Results suggest that change agents perceive the relative advantage associated with two-stage ditches to be low, and that two-stage ditches might be perceived by potential adopters to be incompatible with the prevalent sociocultural beliefs about drainage ditch management. Results also indicate that change agents’ perceptions of environmental benefits of adopting two-stage ditches affects their willingness to promote them. Results are more broadly informative about promoting conservation practices, and is relevant for both academicians and practitioners.

## Introduction & rationale

An innovation is defined as “an idea, practice, or object that is perceived as new by an individual or other unit of adoption” [1]. Conservation practices are a classic, oft studied, example of innovations designed to alleviate problems associated with nonpoint source pollution from agricultural runoff [2]. Decades of scholarly work on adoption of conservation practices, grounded in the diffusion of innovations literature, has examined characteristics of three primary sets of variables: the adopter, the change agent, and the innovation.

First, much scholarly work has focused on identifying factors that lead farmers to adopt conservation practices [3,4]. A review of thirty-five years of quantitative literature on adoption of conservation practices found that, among others, farmers’ seeking and using information, and their awareness of conservation practices, positively influenced adoption [5]. Similarly, in another review of the adoption literature, access to and quality of information, and being connected to agency or local networks of farmers or watershed groups, were identified as variables having the largest impact on adoption [4]. Overall, although literature focusing on adopters of conservation practices has grown immensely over the past few decades, debates have also continued about the inconclusive nature of these findings [6,7].

Second, some scholars have focused on the role of change agents in promoting and explaining adoption [8]. A change agent is “an individual who influences clients’ innovation-decisions in a direction deemed desirable by a change agency” [1, pp. 27]. Studies of change agents, focusing on their individual attributes, have identified several factors important for promoting adoption of innovations, such as, change agents’ authority and technical expertise, perceived similarity to the potential adopters, residence within same community, communication skills, personal relationships with potential adopters, and empathy with the circumstances and problems of potential adopters [1,9]. Other scholars have identified the persuasiveness of change agents being an important factor in explaining adoption of innovations by farmers [8].

Finally, some scholars have examined attributes of the conservation practice itself, which in turn may affect its adoption by farmers [10,11]. Diffusion of innovations literature identifies the following five perceived attributes of innovations: (1) Relative advantage, (2) Compatibility, (3) Complexity, (4) Trialability, and (5) Observability [1; pp. 222]. Specifically, for conservation practices, perceived high levels of relative advantage, compatibility and observability have been identified as being most important in increasing adoption [10]. Other scholars, in addition to emphasizing the importance of perceived high relative advantage, have also identified trialability of conservation practices as being important in increasing adoption [9].

Although much has been written about innovations in irrigation systems [12,13], little is known about innovations in drainage systems. As we already know from the diffusion of innovations theory, change agents play a crucial role by acting as a facilitator of the flow of innovations to potential adopters [1]. However, what we don’t know is what makes change agents want to promote a particular innovation. The objective of this study is to examine change agents’ perceptions of the attributes of two-stage ditches, an innovative agricultural drainage ditch design, and examine what affects their willingness to promote it. The overarching questions of this study are: (1) How do change agents perceive the attributes of two-stage ditches? and (2) What factors influence change agents’ willingness to promote two-stage ditches? The data for this study come from semi-structured interviews with change agents.

### Role of change agents in diffusion of innovations

One of the key variables determining the rate of adoption of innovations in the classic diffusion literature is the extent of change agents’ promotional efforts [1]. Change agents may refer to a wide variety of actors who promote adoption of innovations by providing farmers with, or giving them access to information and inputs which help them adopt an innovation [8]. Change agents have been broadly conceptualized as advisors, agents of state, purveyors of expert knowledge, etc. [14]. Specifically, in the context of agricultural watershed management, change agents are considered to be part of the policy networks which help spread information about conservation practices [2]. In the Western Lake Erie Basin (WLEB) region of Ohio, these individuals include government agents involved closely with advising farmers/landowners about their drainage management practices. Among others, this advice pertains to maintenance of drainage ditches, a practice that has implications for water conveyance, nutrient retention, and biodiversity conservation [15]. Given their involvement in decisions related to drainage management, change agents play a crucial role in promoting two-stage ditches.

There is a widespread scholarly recognition of the crucial role played by change agents in promoting adoption of innovations. For example, knowledge exchange between agronomists (a type of change agent) and farmers, underpinned by trust, credibility, empathy, and consultation, promotes adoption of conservation practices [14]. In another study, the persuasiveness of change agents was found to be an important factor in adoption of innovations by farmers [8]. The crucial role of change agents in establishing the relative advantage of an innovation is also recognized in literature [1]. Scholars have also studied change agents’ views about barriers to adoption of sustainable agricultural practices in southern United States [16]. In a more recent study of drainage professionals in Indiana, a group of change agents, scholars examined their attitudes and characteristics in order to assess their awareness of water quality impairments and conservation practices, and their perceptions of the importance of water quality [17]. However, the extant scholarly work lacks an explicit understanding of factors which make change agents want to promote innovations. As mentioned earlier, the objective of this study is to examine change agents’ perceptions of the attributes of two-stage ditches and examine what affects their willingness to promote them.

### Study context

Ohio’s first drainage laws were passed in 1841 [18]. These laws granted local offices the authority to design and construct drainage projects and assess the costs to the benefitting landowners [19]. Under the auspices of these laws, drainage projects allowed the development of marginal and poor lands intro productive agricultural lands. Part of such a radical transformation of the agricultural landscape has been attributed to the practice of channelization, also called “ditching” [20]. Channelization was typically carried out by creating a “trapezoidal drainage ditch design”, which successfully and efficiently drained the soil profile, but due to its deviations from a natural stream condition, often required “drainage improvements” in order to maintain the design, prevent sediment accumulation, and remove woody vegetation along the ditch banks [20]. In a survey of 47 county drainage programs in Ohio, scholars were able to identify maintenance expenditures in excess of $2.8M (USD) in 1996 [21]. It is also estimated that over 60% or approximately 7 million acres of Ohio’s cropland has drainage improvement [19].

**Fig 1:**
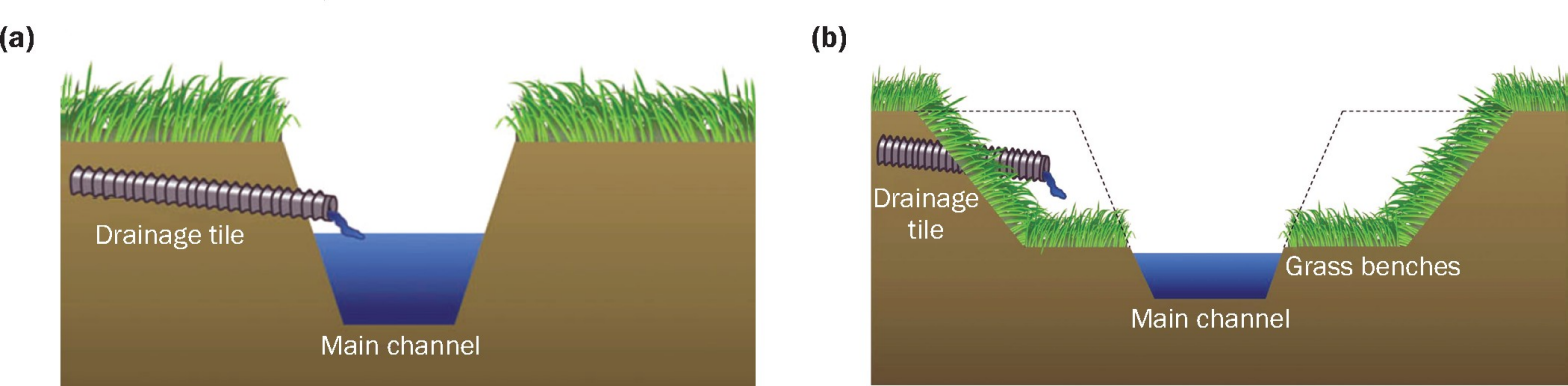
Schematic comparing (a) conventional trapezoidal ditch design and (b) two-stage ditch design [23].

The two-stage ditch is an innovative drainage ditch design which contrasts with the traditional trapezoidal structure of drainage ditches (Fig 1). The traditional trapezoidal design was questioned by researchers in Ohio, leading to the development of the innovative two-stage ditch design, as an alternative to traditional ditch maintenance for the purpose of increasing ditch stability, reducing bank erosion and flooding into adjacent fields [20,22]. The first two-stage ditch was constructed in Wood County, Ohio, in 2002 [20]. The practice has been frequently adopted throughout the westernmost counties in the WLEB and occasionally in pockets within the remainder of the watershed. The two-stage ditch is a floodplain establishment design that includes a narrow inset channel and adjacent floodplains (i.e. the first stage) within the existing ditch. This channel form improves sediment transport during low to intermediate flow periods and reduces sediment accumulation in the ditch bottom. The small floodplains within the ditch increase interactions at the soil-water-vegetation interface, which promotes sediment and nutrient removal [20]. Above the floodplain elevation (i.e. the second stage), the two-stage design is typically wider than a conventional trapezoidal ditch and can handle larger volumes of drainage water. The underlying principle behind two-stage ditches is to incorporate key characteristics of a natural channel system (i.e. floodplains) into the drainage ditch design in order to more closely mimic a natural stream environment [22]. A wide range of scholarly work supports the following non-exhaustive list of benefits associated with two-stage ditches: improves bank stability, maintains or improves drainage capacity, reduces the need for ditch clean-outs, improves habitat for aquatic wildlife, and that the improved soil-water-vegetation interactions may have implications for water quality and ecological benefits [19,21–23].

However, there are also tradeoffs associated with their adoption. For example, since two-stage ditches are generally 10 to 20 ft. wider than trapezoidal drainage ditches, there is a loss of 1 to 3 acres of land per linear mile of two-stage ditch [22]. In addition to the loss of land, the initial excavation and construction costs of two-stage ditches are also greater than excavation costs of trapezoidal drainage ditches [22]. Furthermore, challenges have arisen where two-stage ditches are adopted at sites with grass filter strips already enrolled in federal conservation programs (e.g. Conservation Reserve Program) and construction encroaches on the filter area receiving a cost-share land rental payment. In some instances, landowners have incurred fines, had to reimburse past conservation payments, been required to expand an existing filter strip to compensate for losses related to two-stage ditch construction, or forfeited future payments when an enrolled filter strip is affected [20]. There are also concerns of potential downstream flooding since two-stage ditches increase flow-handling capacity by 25% to 100% [22].

### Research method & data collection

This study was approved by the Behavioral Institutional Review Board at The Ohio State University (IRB Protocol Number: 2013B0509). Oral consent was obtained from study participants.

The data for this study come from semi-structured interviews with government agents. This exploratory study was conducted in the Western Lake Erie Basin region of Ohio. The phone interview data was obtained from one government agent each in 17 counties across the Western Lake Erie Basin. Interviews were not conducted with the goal of reaching a point of saturation. Instead, the goal was to conduct one interview in each county, with an interviewee who was knowledgeable about two-stage ditches. The criteria for selection of an interviewee was based on their involvement in drainage improvement and maintenance in a county. Since advising landowners about drainage ditch management is one of the main responsibilities of these interviewees, it is important to understand their perception about two-stage ditches. All the interviewees in this study were asked if they were aware of two-stage ditches and would be willing to be interviewed on this topic. Ten out of 17 interviewees were from Soil & Water Conservation District (SWCD) offices. The remaining seven interviewees were from County Engineers’ Offices (CEOs). Some of the most common interviewee designations were: district technicians, drainage engineer, ditch maintenance supervisor, and county engineer. All our interviewees were male, and their experience with respect to advising about agricultural drainage and other conservation practices ranged from a year to 45 years. Fifteen phone interviews were conducted during September, 2013 to December, 2013; two respondents sent responses by email. The interview duration ranged from 60 minutes to 75 minutes. All the interviews were then transcribed.

Interviews in this study included questions about what interviewees thought about two-stage ditches as a management practice, their perception about whether or not they are beneficial and why, what promotes and hinders adoption of two-stage ditches, and whether and why they thought farmers would be willing or not willing to adopt two-stage ditches. Although the focus of the interview was on identifying perceived relative advantage of adopting two-stage ditches, the emerging themes from the interviewee data were situated more broadly within the five perceived attributes of innovations (relative advantage, compatibility, complexity, trialability, and observability) [1]. The underlying goal of this analysis was to understand the attributes of two-stage ditches. Systematic qualitative techniques such as coding, pattern matching, and synthesis were used to analyze the collected data. Codes for this study were developed inductively, as a result of direct examination of the interview data. Quotes and short vignettes were instructive. This allowed for synthesis of interpretations and identification of themes that cut across interviewees [24]. Data analysis validity was achieved by utilizing the process of ‘constant comparison’, which involved reading and re-reading interviews to search for and identify emerging themes and by having data validated by a few interviewees, also known as respondent validation or member check [25,26]. Data analysis was conducted using MAXQDA (a qualitative data analysis software; version 11).

## Results

The results section in this study is split into several sections. The first two sections focus on change agents’ perceived relative advantages/disadvantages, and tangibility and immediacy of benefits associated with two-stage ditches. The next two sections focus on perceived attributes of “compatibility” and “complexity” of innovations. Although diffusion of innovations literature identifies five perceived attributes of innovations, trialability and observability did not emerge as recurring themes in this study. The following section synthesizes across multiple perceived attributes of two-stage ditches and examines their effect on change agents’ willingness to promote them.

### Perceptions of relative advantages/disadvantages

Relative advantage indicates the costs and benefits resulting from adoption of an innovation [1]. Interviewees in this study identified a range of benefits/advantages associated with the adoption of two-stage ditches (Table 1).

**Table 1:** Advantages associated with two-stage ditch.

Benefit/Advantage	Frequency^a^	Percentage^b^ (n = 17)
Water quality benefits	8	7 (41%)
Less/No future maintenance cost; Self cleaning	10	7 (41%)
Flood relief/control; act as retention/detention pond	8	6 (35%)
Less sediment runoff/reduces sedimentation	3	3 (18%)
Stable ditch banks	3	3 (18%)
Easy to maintain	1	1 (<6%)
Increases ditches’ longevity	1	1 (<6%)

^a^ Includes all mentions across all interviewees (e.g., if an interviewee mentioned an advantage twice, it was counted as two times in the frequency column).

^b^ Number of all interviewees who mentioned an advantage at least once.

The greatest number of interviewees, seven out of 17, identified water quality benefits and the benefit of less to no future maintenance costs associated with two-stage ditches. An important point to keep in mind is that four out of 17 interviewees did not identify any benefits associated with two-stage ditches. Instead, they identified disadvantages associated with them. Although a fewer number of disadvantages (five disadvantages vs seven advantages) associated with two-stage ditches were identified, the number of interviewees who identified these disadvantages, as well as the frequency with which they were identified, was much higher when compared with advantages associated with them (Table 2).

**Table 2:** Disadvantages associated with two-stage ditch.

Non-Benefit/Disadvantage	Frequency^a^	Percentage^b^ (n = 17)
Permanent loss of farming land	30	16 (94%)
Increased cost of construction	13	10 (59%)
Future maintenance difficult	5	5 (29%)
Issue of dealing with excavated soil	4	2 (12%)
Context specific–Trees cut for construction; against environment	1	1 (<6%)

^a^ Includes all mentions across all interviewees (e.g., if an interviewee mentioned a disadvantage twice, it was counted as two times in the frequency column).

^b^ Number of all interviewees who mentioned a disadvantage at least once.

The greatest number of interviewees, 16 out of 17, identified that adoption of two-stage ditch would require permanent loss of farming land. This was followed by the disadvantage of increase in the cost of construction of two-stage ditches, especially in comparison with the cost associated with construction of traditional drainage ditches. For example, an interviewee mentioned, “Two-stage ditches are more expensive than conventional drainage ditch designs”. Another interviewee who emailed his response, wrote, “I believe it would be safe to estimate that a two-stage channel could cost double that of a standard design for an annual storm”. This was followed by the disadvantage of difficulty with future maintenance of two-stage ditches. Highlighting this disadvantage, one of the interviewees mentioned, “The two-stage ditches are so wide that typical [maintenance] equipment couldn't reach the center [of the ditch] making maintenance a nightmare”. Another interviewee added, “An issue with two-stage ditch is when you have to remove the sediments [carry out a form of ditch maintenance]. Since the top has been widened it becomes tougher to clean it”. This was followed by, in decreasing order, dealing with the soil that was excavated in order to construct them two-stage ditch, and the context specific factor that sometimes trees are cut in order to construct these ditches, which goes against the environmental benefits associated with them.

As the above analysis indicates, adoption of two-stage ditches involves incurring immediate costs in the form of losing land and incurring high initial costs, whereas the benefits are distant in the form of water quality benefits, reduction in future ditch maintenance costs, and flood relief. Talking about these tradeoffs, an interviewee mentioned, “With two-stage ditches, the economic offset is not enough for me to be able to go and promote it”. They further added, “We have had meetings about two-stage ditches, more than anything, it is the cost that kills it”. Overall, given the balance of evidence, it can be argued that the “relative advantage” associated with two-stage ditches is low. As per Rogers (2003), the low perceived relative advantage of an innovation is also due to the fact that rewards from adopting them are relatively intangible and often delayed in time.

### Intangibility & immediacy of benefits

Interviewees in this study identified several dimensions of intangibility of benefits associated with the adoption of two-stage ditches. These dimensions of intangibility of benefits are: temporal, uncertainty, economic, spatial, and functional (Table 3).

**Table 3:** Dimensions of intangibility of benefits associated with two-stage ditch.

Dimensions of intangibility of benefits	Frequency^a^	Percentage ^b^ (n = 17)
Temporal	10	9 (53%)
Uncertainty	7	6 (35%)
Economic	3	3 (18%)
Spatial	2	2 (12%)
Functional	2	1 (<6%)

^a^ Includes all mentions across all interviewees (e.g., if an interviewee mentioned a dimension twice, it was counted as two times in the frequency column).

^b^ Number of all interviewees who mentioned a dimension of intangibility at least once.

The greatest number of interviewees, nine out of 17, identified the temporal dimension of intangibility of benefits associated with adoption of two-stage ditches. Although the frequency of any single dimension of intangibility of benefits associated with two-stage ditches is low, it is important to note that several interviewees mentioned temporal and uncertainty dimensions of intangibility of benefits associated with adoption of two-stage ditches. Unlike the temporal dimension–an assessment of intangibility of benefits with respect of immediate costs and delayed benefits, uncertainty of benefits pertained to the benefits themselves being uncertain.

Temporal intangibility of benefits was identified by interviewees from the perspective that although costs associated with adoption of two-stage ditches are immediate, the benefits are delayed in time. For example, an interviewee emphasizing the aspect of immediate costs and delayed benefits, mentioned, “Up here in our county, a landowner won’t see any benefit associated with a two-stage ditch as he is losing land”. Another interviewee mentioned, “They [landowners] do not see the benefits for the extra costs in building huge two-stage channels [two-stage ditches].” The aspect of intangibility of long-term benefits was captured very well by an interviewee, who mentioned, “Landowners do not see the long-term benefits associated with two-stage ditches”.

The intangibility dimension of uncertainty of benefits was identified by interviewees with respect to the benefits being difficult to perceive and demonstrate. For example, an interviewee mentioned, “As of now, I am at 50–50 level in terms of whether they [two-stage ditches] are beneficial or not. I know that they are projected not to have any maintenance requirement…but are these benefits going to last?” Another interviewee mentioned, “Farmers do not see tangible benefits associated with adopting two-stage ditch…It is hard to demonstrate benefits of a two-stage ditch [to farmers]”.

Economic intangibility of benefits was identified by interviewees from the perspective that attributing an economic value to the benefits associated with two-stage ditches is difficult. For example, an interviewee mentioned, “They [landowners] are not willing [to adopt two-stage ditch] because they don’t see any tangible economic benefit”. Another interviewee mentioned, “Farmers are mainly interested in and more willing for BMPs that benefits/pays them. If the BMP [referring to two-stage ditch] lacks a tangible benefit then they are not willing to go for it. Economics comes into play”. Another interviewee further added, “How do we put a dollar value on the benefits of two-stage ditches?”

Spatial intangibility was identified by interviewees from the perspective of the location of benefitting landowners in the watershed with respect to the two-stage ditch. This aspect was captured very well by an interviewee, who mentioned, “Who is going to receive the benefits due to construction of a two-stage ditch? How far is a farmer from the ditch and how is he being assessed the cost”. Another interviewee, also referring to the aspect of spatial intangibility of benefits of adopting two-stage ditch, mentioned, “Most of the people [landowners] are not right next to a drainage ditch; only 10% are next to a drainage ditch”. The interviewee was indicating that since a majority of landowners in the watershed are spatially away from the drainage ditch, they will not perceive that two-stage ditches are beneficial.

Functional intangibility dimension pertains to the notion that adoption of two-stage ditches does not lead to any perceptible change in the flow of drained water–the primary function of drainage ditches in an agricultural landscape. This dimension relates closely with Roger’s (2003) finding that the rate of adoption of a new innovation is affected by the old innovation that it supersedes. The yardstick against which an innovation is compared is the practice that it supersedes. As mentioned earlier, the goal with the traditional trapezoidal ditch design was to successfully and efficiently drain the soil profile. However, with the adoption of the two-stage ditch design, as reported by an interviewee in this study, farmers do not see any perceptible change in the flow of drained water. They mentioned, “Farmers don’t see any tangible benefit [from adoption of two-stage ditches] as they don’t see any increase in water flow”.

As the above analysis indicates, the benefits associated with adoption of two-stage ditches are indeed intangible, often delayed in time, and are sometimes tentative.

### Compatibility of the innovation

In addition to the perceived relative advantage of two-stage ditches, the next important theme that emerged from interviewee data was that of “compatibility”. Compatibility of an innovation refers to the degree to which an innovation is perceived as being consistent with existing values, past experiences, and needs of potential adopters [1; pp. 240]. An innovation can be compatible or incompatible with respect to need, previously introduced ideas, or sociocultural values and beliefs [1]. Interviewees in this study identified several dimensions pertaining specifically to incompatibility of two-stage ditches with needs of potential adopters (Table 4).

**Table 4:** Dimensions of incompatibility with needs associated with two-stage ditch.

Dimensions of incompatibility with needs	Frequency^a^	Percentage^b^ (n = 17)
Perception of there not being a need for the benefit provided by two-stage ditch	6	4 (24%)
No current issues with drainage ditches	2	2 (12%)
Perception that two-stage ditch won’t fix the existing issues	2	2 (12%)
Old design does what two-stage ditch design promises	2	2 (12%)

^a^ Includes all mentions across all interviewees (e.g., if an interviewee mentioned a dimension of incompatibility with needs twice, it was counted as two times in the frequency column).

^b^ Number of all interviewees who mentioned a dimension of incompatibility with needs at least once.

Several interviewees, four out of 17, perceived that there was a lack of need for an increased drainage capacity provided by two-stage ditches. For example, an interviewee mentioned, “Our ditches are already functioning with the [drainage] capacity as a two-stage [ditch] would make it function. We already have a similar process going on which is as good as two-stage ditches”. Another interviewee added, “Currently they [landowners] do not see a need to make a ditch bigger in size [to obtain an increased drainage capacity] than what they already have”. Further insights about the perception that there are no issues with existing drainage ditches and hence there isn’t a need to change it, was provided by two interviewees. One of them mentioned, “If they [landowners] don’t have a problem with integrity/stability of bank, then why should they go for a two-stage ditch?” Another interviewee added, “The ditches that we have in our county are very old and they have been working very well. So, the notion is why to change something that has been working fairly well”.

Evidence about the perception that two-stage ditches won’t fix drainage issues in areas with flat-terrain and excessive sedimentation, was provided by two different interviewees. One of them mentioned, “The downfall that I can see in my county [with respect to the benefit of adoption of two-stage ditch] is that we are very flat up here. Our main priority here is to have our tile outlet”. The second interviewee mentioned, “In our county, long-term ditch maintenance benefit does not come into picture because of loads of sedimentation. Irrespective of the drainage design [whether two-stage ditch or trapezoidal ditch], sedimentation becomes the main issue, as it blocks the outlet [of drainage] tiles”. Both the interviewees implied that the benefits provided by two-stage ditches are not compatible with the local drainage ditch maintenance needs.

Evidence that existing ditch designs already mimic the conditions and provides water quality benefits was provided by an interviewee, who mentioned, “In our county we have more naturally forming ditches. We have canary grasses here which have a tendency to filter the water anyways. Thus, I don’t see any benefit of promoting a two-stage ditch”. Another interviewee felt that the existing design is already self-cleaning, and thus provides the benefits which two-stage ditches promise. They mentioned, “I have channels [drainage ditches] under maintenance for over 25 years that have never had to have the bottoms excavated [a form of ditch maintenance] because they were designed at the optimum width for the annual storm and keep themselves clean”.

Moreover, evidence was found in support of incompatibility of two-stage ditches with previously introduced ideas, and sociocultural values and beliefs–two additional dimensions of incompatibility identified in the diffusion of innovations literature [1]. As per Rogers (2003), previous practice provides a familiar standard against which an innovation is compared and interpreted. Three interviewees provided evidence in support of incompatibility of two-stage ditches with previously introduced idea. The first interviewee mentioned, “A lot of ditches in my county are pretty small, wherein the bottom is at the most two feet wide. So, a two-stage ditch won’t fit there”. The second interviewee mentioned, “We have low gradient ditches, so two-stage ditches won’t be a practical solution in our county”. The third interviewee mentioned, “We pretty much have a standard ditch design here. There is not much here that we can change”.

Two interviewees provided evidence in support of incompatibility of two-stage ditches with existing sociocultural values and beliefs about drainage ditches. The first interviewee mentioned, “A lot of ditches have been built years ago, farmers want them to be built back to the original [trapezoidal] design”. Highlighting the predominant notion of draining water, he further added, “Landowners care more about their water. They want their water to go away [get drained]”. The second interviewee mentioned, “I think mind-set acts as a hindering factor. There is this mind-set of doing things in the traditional way. It goes back to the old generation thinking, if it worked then why change”. In this study, reluctance to change is due in part to the two-stage ditches’ incompatibility with the sociocultural beliefs of potential adopters.

### Complexity of the innovation

In addition to the perceived relative advantage and compatibility of two-stage ditches, the next important theme that emerged from interviewee data was that of “complexity”. Complexity of an innovation refers to the degree to which an innovation is perceived as relatively difficult to understand and use [1]. In this study, complexity of two-stage ditches has been conceptualized from the dimension of their “conditional suitability”, which implies that the many elements of local conditions have to be right for two-stage ditches to be applicable and useful. Rather than being equally applicable everywhere, two stage-ditch suitability depends on a number of interacting contextual factors. Understanding the conditional suitability of two-stage ditches is important for their promotion, highlighted by an interviewee, who mentioned, “If we found a place where a two-stage ditch was feasible and practical, then we [change agents] can promote it”. While some of the interviewees did not identify the specific conditions in which two-stage ditches’ would be suitable, others felt that their suitability was driven by topographical features such as floodplain, type of soil, land gradation, and the level of urbanization in an area. Another interviewee felt that they are suitable if only there is a need for greater drainage capacity (Table 5).

**Table 5:** Dimensions of complexity associated with two-stage ditch.

Dimensions of Complexity as a function of conditional suitability	Frequency^a^	Percentage^b^ (n = 17)
General conditional suitability	5	5 (29%)
Suitability driven by topography	5	4 (24%)

^a^ Includes all mentions across all interviewees (e.g., if an interviewee mentioned a dimension of complexity twice, it was counted as two times in the frequency column).

^b^ Number of all interviewees who mentioned a dimension of complexity at least once.

The general conditional suitability of two-stage ditches was highlighted by an interviewee, who mentioned, “The location, and condition has to be right. Two-stage ditches cannot be incorporated into every project. I am not an expert, but from my experience, the conditions have to be good for two-stage ditches to be beneficial”. Another interviewee added, “I think in certain areas two-stage ditches would be more adoptable compared with other ditch designs. Whereas, there are places where a conventional ditch does the job. There is a fine line when it comes to where a two-stage ditch can work, and where it can’t”. Although these interviewees did not identify specific conditions, their response highlights the fact that two-stage ditches are complex in terms of the conditions under which they can be applicable and useful.

Another important dimension of complexity associated with two-stage ditches is that their suitability is driven by topographical features, which implies that topographical conditions have to be right for two-stage ditches to be applicable and useful. An interviewee who felt that two-stage ditches are suitable in a floodplain area, mentioned, “When you are almost into a floodplain, this could be the area where you could construct a two-stage ditch”. They further added, “A two-stage ditch would work well if you are in a region which is more of a natural creek, and you need better flow in order to avoid flooding issues”. Another interviewee highlighting the importance of soil type in determining two-stage ditches’ suitability, mentioned, “Soil type is an important driving force when it comes to deciding about a ditch design. If the soil type is sandy, then a two-stage design would work better as it helps in widening the ditch and providing bank stability”. An interviewee who felt that land gradation was an important topographical feature in determining suitability of two-stage ditches, mentioned, “I don’t think two-stage ditches are the answer on a flat [land] grade. I think they would be beneficial if there is enough [land] gradation [slope]”. Another interviewee who felt that two-stage ditches will not be feasible in urbanized areas, mentioned, “There are residential areas where it is not possible to do a two-stage ditch …due to zoning/building regulations”.

The above analysis provides evidence for complexity of two-stage ditches as a function of its conditional suitability, which means that many elements of local conditions have to be right for two-stage ditches to be a viable innovation.

### Willingness to promote two-stage ditches

While describing the variety of perceived attributes of two-stage ditches is instructive, an important question remains: how do change agents’ perceived attributes of two-stage ditches affect their willingness to promote them? To synthesize across multiple perceived attributes of two-stage ditches, and examine their effect on willingness to promote, interviewees were arranged in decreasing order of their willingness to promote two-stage ditches (Table 6).

**Table 6:** Perceived attributes & willingness to promote two-stage ditches.

Code^a^	Type of Benefits							Type of Disadvantages					Incompatible^b^^,^^c^	Complex^c^	Willingness to promote^d^
	B1	B2	B3	B4	B5	B6	B7	D1	D2	D3	D4	D5			
A6	**✓**	**✓**	**✓**	✘	✘	✘	✘	**✓**	**✓**	✘	✘	✘	**✓**	**✓**	8.5
A7	**✓**	✘	**✓**	**✓**	**✓**	✘	**✓**	**✓**	**✓**	✘	✘	✘		**✓**	8
A1	✘	**✓**	✘	✘	✘	✘	✘	**✓**	**✓**	**✓**	✘	✘	**✓**	**✓**	7
A13	**✓**	**✓**	✘	**✓**	✘	✘	✘	**✓**	✘	✘	✘	✘	**✓**		7
A11	✘	**✓**	✘	**✓**	✘	✘	✘	✘	**✓**	✘	**✓**	✘			7
A16	**✓**	**✓**	**✓**	✘	**✓**	✘	✘	**✓**	✘	✘	✘	✘			5.5
A12	**✓**	✘	✘	✘	✘	✘	✘	**✓**	✘	✘	✘	✘	**✓**	**✓**	5
A8	✘	✘	**✓**	✘	✘	✘	✘	**✓**	**✓**	✘	✘	✘			4
A9	✘	✘	✘	✘	✘	✘	✘	**✓**	**✓**	✘	**✓**	✘	**✓**	**✓**	4
A3	✘	✘	✘	✘	✘	✘	✘	**✓**	✘	**✓**	✘	**✓**	**✓**	**✓**	4
A5	✘	✘	**✓**	✘	✘	✘	✘	**✓**	**✓**	**✓**	✘	✘			3.5
A17	✘	**✓**	✘	✘	✘	✘	✘	**✓**	✘	✘	✘	✘			3
A4	**✓**	**✓**	✘	✘	**✓**	**✓**	✘	**✓**	✘	✘	✘	✘			2.5
A2	**✓**	✘	✘	✘	✘	✘	✘	**✓**	**✓**	✘	✘	✘	**✓**	**✓**	2
A14	✘	✘	✘	✘	✘	✘	✘	**✓**	✘	✘	✘	✘	**✓**		1
A15	✘	✘	✘	✘	✘	✘	✘	**✓**	**✓**	**✓**	✘	✘			1

A ‘**✓**’ mark indicates that a particular benefit/advantage was mentioned by an interviewee. A ‘**✘**’ mark indicates that a particular benefit/disadvantage was not mentioned by an interviewee.

**BENEFITS–B1**: Water quality benefits; **B2**: Less/no future maintenance cost; **B3**: Flood relief/control; **B4**: Reduces sedimentation; **B5**: Stabilizes ditch bank; **B6**: Easy to maintain; **B7**: Increases ditches’ longevity

**DISADVANTAGES–D1**: Loss of land; **D2**: Increased cost of construction; **D3**: Future maintenance difficult; **D4**: Dealing with excavated soil

**D5**: Environmental costs (trees cut for construction).

^a^ Refers to the code assigned to an interviewee in this study. Although 17 interviews were conducted as part of this study, one interviewee did not provide data on his willingness to promote two-stage ditch, and has been excluded from this analysis.

^b^ Incompatibility measure includes incompatibility with needs dimensions, as well as incompatibility with existing drainage ditch design and incompatibility with sociocultural beliefs about drainage ditch management goals.

^c^ Blank cells in this column indicate that this theme was not identified by an interviewee.

^d^ Willingness to promote two-stage ditches was measured on a scale of 1 to 10, with 1 being not at all willing and 10 being very willing.

Analysis reveals several patterns suggesting which variables might be influential in determining outcomes. The set of 12 benefits and disadvantages listed in the table represent the “perceived relative advantage” of two-stage ditches. Incompatibility and complexity are two additional themes listed in the table. Together, these variables represent the perceived attributes of two-stage ditches, which are the independent variables, while willingness to promote is the dependent variable.

The highlighted section of the table groups the seven respondents who indicated a willingness to adopt of at least the midpoint of the scale (5). These scores ranged from 5 to 8.5 with a mean value of 6.86. The non-highlighted section groups the nine respondents who indicated a willingness to adopt below the midpoint of the scale (5). These scores ranged from 1 to 4 with a mean value of 2.78. A count of check marks under ‘types of benefits’ reveal that benefits were identified 19 times by the seven interviewees in the highlighted section of the table. On the contrary, in the non-highlighted section, benefits are identified only eight times by nine interviewees. In fact, four out of these nine interviewees (A9; A3; A14 & A15) did not identify any benefit associated with two-stage ditches. A similar count of check marks under ‘types of disadvantages’ reveals that disadvantages were identified 12 times by the seven interviewees in the highlighted section of the table. On the contrary, in the non-highlighted section, disadvantages were identified 19 times by nine interviewees. Specifically, 19 benefits versus 12 disadvantages (high perceived relative advantage) for interviewees in the highlighted section of the table, is associated with higher mean willingness (*M*) to promote (*M* = 6.86; n = 7). Similarly, eight benefits versus 19 disadvantages (low perceived relative advantage) for interviewees in the non-highlighted section of the table, is associated with lower mean willingness to promote (*M* = 2.78; n = 9). Broadly, this analysis reveals that perceived relative advantage is associated with willingness to promote.

Considering specific benefits, one at a time, while comparing across interviewees in the highlighted (with high willingness scores) and non-highlighted (with low willingness) sections of Table 6, seven out of 16 interviewees identified water quality benefits (B1), five in the highlighted section of the table and two in the non-highlighted section. Identifying water quality benefits reflects an interviewees’ recognition that two-stage ditches have environmental benefits, a critical selling point for conservation practices. The five interviewees in the highlighted section of the table also had high willingness to promote two-stage ditch, which indicates that perceptions of environmental benefits is important for change agents to be willing. We would have expected the two interviewees (A4 & A2) in the non-highlighted section to also have high willingness. For interviewee A4, who also listed three other benefits (B2, B5 & B6), their willingness to promote was affected by their perception of low acceptability of two-stage ditches by landowners in their county. Interviewee A2, who identified water quality benefits as the only advantage, felt that the economic offset, given the loss of land (D1) and increased cost of construction (D2), was not enough for them to promote two-stage ditches. Despite these two outliers, it can be concluded that perceptions of environmental benefits is important for change agents to be willing to promote two-stage ditches.

For the benefit of less or no future maintenance cost (B2), seven out of 16 interviewees identified this benefit, five in the highlighted section of the table and two in the non-highlighted section. Given that recurring, annual maintenance cost is an important aspect of ditch maintenance, identification of this benefit by interviewees is indicative of their recognition that adoption of two-stage ditches can reduce/remove this cost in the future. The five interviewees in the highlighted section of the table also had a high willingness to promote two-stage ditch, which indicates that perceptions of less or no future maintenance cost is important for change agents to be willing. We would have expected the two interviewees (A17 & A4) in the non-highlighted section to also have high willingness. For A4, as explained earlier, his willingness to promote was affected by his perception of low acceptability of two-stage ditches by landowners in his county. Interviewee A17 felt that his organizations’ lack of knowledge would necessitate seeking outside help to implement two-stage ditches. Despite these two outliers, it can be concluded that perception of less or no future maintenance cost is important for change agents to be willing to promote two-stage ditches.

For flood relief/control (B3), five out of 16 interviewees identified this benefit, three in the highlighted section of the table and two in the non-highlighted section. Since almost equal number of interviewees (three vs two) identified this benefit on both sides of the willingness to promote spectrum, this factor is not a driver in affecting change agents’ willingness to promote two-stage ditches. Three out of 16 interviewees identified reduction of sedimentation as a benefit (B4), all of them in the highlighted section of the table. These interviewees also had a high willingness to promote two-stage ditch, which indicates that perceptions of reduction in sedimentation is important for change agents to be willing to promote two-stage ditches. Stabilization of ditch banks (B5) was identified as a benefit by three out of 16 interviewees, two in the highlighted section of the table and one in the non-highlighted section. Since almost equal number of interviewees (two vs one) identified this benefit on both sides of the willingness to promote spectrum, this factor is not a driver in affecting change agents’ willingness to promote two-stage ditches. The remaining two benefits (B6 & B7), were identified only once, and were therefore not considered as drivers in affecting change agents’ willingness to promote two-stage ditch.

Looking more specifically at disadvantages, 15 out of 16 interviewees identified loss of land (D1), six in the highlighted section of the table and nine in the non-highlighted section. Except for interviewee A11, every interviewee identified the disadvantage of loss of land associated. Hence, although this is an important disadvantage, it is not driving change agents’ willingness to promote two-stage ditch. Considering the disadvantage of increased cost of construction (D2), nine out of 16 interviewees identified this disadvantage, four in the highlighted section of the table and five in the non-highlighted section. Since almost equal number of interviewees (four vs five) identified this benefit on both sides of the willingness to promote spectrum, this factor is not a driver in affecting change agents’ willingness to promote two-stage ditch.

The disadvantage that future maintenance may become difficult with a two-stage ditch design (D3) was identified by four out of 16 interviewees, one in the highlighted section of the table and three in the non-highlighted section. An interviewee identifying this disadvantage reflects his apprehension of not only the difficulty in maintaining two-stage ditches in the future, but also his disagreement with the underlying principle that the two-stage ditches are self-cleaning. The three interviewees in the non-highlighted section of the table also had a low willingness to promote two-stage ditch, which indicates that perceptions of difficulty with future maintenance is a driver in affecting change agents’ non-willingness to promote two-stage ditch. We would have expected interviewee A1 in the highlighted section to also have low willingness. However, it is important to note that unlike other interviewees (A3, A5, & A15) who did not identify the benefit of less to no future maintenance cost (B2), interviewee A1 recognizes this benefit, which, as identified earlier is an important driver in affecting change agents’ willingness to promote two-stage ditch. Despite an outlier, it can be concluded that perception of difficulty with future maintenance is important in affecting change agents’ non-willingness to promote two-stage ditch.

Considering the disadvantage that adoption of two-stage ditch will involve dealing with soil excavated for its construction (D4), two out of 16 interviewees identified dealing with excavated soil as a disadvantage, one in the highlighted section of the table and one in the non-highlighted section. Since equal number of interviewees identified this disadvantage on both sides of the willingness to promote spectrum, this factor is not a driver in affecting change agents’ willingness to promote two-stage ditch. The disadvantage of environmental costs of adoption of two-stage ditch (D5) was identified only once and, therefore, is not considered as a driver in affecting change agents’ willingness to promote two-stage ditches.

The theme of incompatibility was identified by eight out of 16 interviewees, four in the highlighted section of the table and four in the non-highlighted section. Since equal number of interviewees identified this theme on both sides of the willingness to promote spectrum, it is not a driver in affecting change agents’ willingness to promote two-stage ditches. In terms of complexity, two-stage ditches were conceptualized from the dimension of their “conditional suitability”, which implies that the many elements of local conditions have to be right for two-stage ditches to be viable. The theme of complexity was identified by seven out of 16 interviewees, four in the highlighted section of the table and three in the non-highlighted section. Since almost equal number of interviewees identified this theme on both sides of the willingness to promote spectrum, it is not a driver in affecting change agents’ willingness to promote two-stage ditches.

To sum up, the benefits positively affecting change agents’ willingness to promote two-stage ditch were: water quality benefits, perception of less or no future maintenance cost, and perceptions of reduction in sedimentation. In contrast, perception of difficulty with future maintenance was identified as a disadvantage negatively affecting change agents’ willingness. Overall, perceived relative advantage was found to be associated with change agents’ willingness to promote two-stage ditch. We did not find conclusive evidence for compatibility and complexity driving change agents’ willingness.

## Discussion

Scholarly work on adoption of conservation practices has often examined the characteristics of three sets of variables: the adopter, the change agent, and the innovation. The diffusion of innovations literature clearly establishes the crucial role of change agents in demonstrating the relative advantage of an innovation to potential adopters. However, the existing literature is thin on explaining what makes change agents want to promote a particular innovation. Taking the example of an innovative two-stage ditch design, and examining change agents’ perception of its attributes, leads to theoretical as well as practical insights.

Firstly, the study found that change agents perceive two-stage ditches as low in relative advantage, and that benefits from adopting them are intangible and often delayed in time. Change agents also identified several nuanced dimensions of perceived attributes. Scholarly work highlights the difficulty change agents have in promoting innovations since it is difficult for them to demonstrate their relative advantage to the adopter [1]. However, this study also highlights the importance of demonstrating relative advantage of adopting innovations to change agents themselves. The perceived relative advantage of two-stage ditches is associated with change agents’ willingness to promote them. Thus, perceived relative advantage not only influences the willingness to adopt an innovation, but also the willingness to promote it. Hence, one of the crucial roles of those wishing to encourage innovations should be to influence the change agents’ perception about the relative advantage of adopting an innovation. To that end, both scholars and practitioners could draw upon our current understanding of farmers’ trusted information sources to seek potential avenues to influence change agents, such as, university extension and watershed groups [27,28].

Secondly, the study found that willingness is driven down by disadvantages like perceived difficulty of future maintenance, while it is driven up by benefits specific to drainage (e.g., less or no future maintenance cost and reduction in sedimentation) and to the environment (e.g., water quality benefits). This finding corroborates scholarly work arguing that the role of agricultural drainage is shifting from that of water conveyance, to a more holistic approach which also takes into account environmental benefits [22,29]. However, it is also important to highlight that several interviewees in this study seemed to misunderstand the purpose of two-stage ditches, since they perceived a lack of need for an increased drainage capacity provided by two-stage ditches (Table 4), without recognizing the water quality benefits associated with them. This finding supports a recent study wherein authors found a tendency among drainage professionals to view the benefit of two-stage ditches as one of maintaining water capacity, instead of improving water quality [17]. Speaking more generally, it is important that change agents do not misunderstand the purpose of innovations they are promoting.

Thirdly, several findings in this study are supported by extant scholarly work. For example, the study found evidence in support of incompatibility of two-stage ditches with existing sociocultural values and beliefs about drainage ditches, in part due to landowners’ reluctance to change. In a study of change agents in southern United States, reluctance to change by farmers was identified as the most frequently mentioned barrier to adoption of sustainable agricultural practices [16]. This study also found evidence in support for incompatibility not being an important factor in having an effect on change agents’ willingness to promote two-stage ditches. This finding is in line with existing literature, which, although focuses on adopters’ perception of compatibility unlike this study’s focus on change agents’ perception of compatibility, finds that compatibility is somewhat less important in predicting rate of adoption than is perceived relative advantage [1]. This study also found evidence in support of complexity not being an important factor in having an effect on change agents’ willingness to promote two-stage ditches. This finding is in line with existing literature, as per which, complexity may not be as important as perceived relative advantage for adoption of innovations [1].

Fourthly, our findings suggest that the relative advantages/disadvantages of the two-stage ditch must be more clearly described or quantified to facilitate decision making of change agents and farmers/landowners. The strongest research on two-stage ditches has focused on water quality, a benefit that change agents mentioned as intangible and delayed. We therefore believe that more research regarding the advantages that would potentially influence farmers/landowners to adopt two-stage ditch when warranted is sorely needed. This might include longer-term studies to support claims of reduced maintenance and enhanced drainage leading to increased yields.

Finally, many of the change agents perceived that they did not prefer a two-stage ditch when the existing ditch was satisfactory. As per two-stage ditches’ design guidance, they should not be implemented if there is not a problem to solve at a site [30]. On sites where there is a problem, the two-stage ditch is just one of six potential solutions that is recommended by the design guidance. Other management options include a “do nothing” approach [31], passive enhancement [31], threshold channel design [32], self-forming channel design [33], and natural channel design [34]. While this highlights the need for educating change agents about two-stage ditches, especially with respect to their suitability in a given context, this also begs the question, how likely would change agents be willing to promote two-stage ditches if they had a better understanding that the two-stage ditch is not recommended as a wholesale substitute for the traditional trapezoidal practice, but on a case by case basis.

## Conclusion

Although research about the importance of change agents in promoting innovations is abundant, we lack an understanding about their perception of attributes of an innovation and how that affects their willingness to promote it. Recognizing this gap, the goal of this study was to examine change agents’ perceptions of the attributes of two-stage ditches. Although perceived “relative advantage”, one of the oft-studied attributes of innovations, provided the starting point of inquiry, the emerging themes from the data were situated more broadly within the five perceived attributes of innovations (relative advantage, compatibility, complexity, trialability, and observability).

Qualitative data analysis from one government agent in each of 17 counties across the Western Lake Erie Basin established that two-stage ditches have low perceived relative advantage, that benefits from adopting them are intangible and delayed in time. Interviewee insights also helped identify several dimensions of intangibility of benefits: temporal, economic, spatial, functional, and uncertainty. Evidence was found in support of two-stage ditches’ incompatibility with needs, incompatibility with existing drainage ditch design, and incompatibility with sociocultural beliefs about drainage ditch management goals. Although diffusion of innovations literature identifies five perceived attributes of innovations, trialability and observability did not emerge as recurring themes in this study. Analysis of perceived relative advantage led to identification of four factors (three positive and one negative) affecting change agents’ willingness to promote two-stage ditches.

Situating this study in the diffusion of innovations literature provided a theoretically rich, as well as conceptually sound foundation for understanding change agents’ perceptions of the attributes of two-stage ditches. However, given our qualitative research design, we cannot make any claims about generalizability of our findings. Nevertheless, our results provide a rich description of change agents’ perceptions of the attributes of two-stage ditches and factors influencing their willingness to promote them. As much as this study provides theoretical and practical insights, it also leaves a number of questions unanswered. In particular, results provide several avenues for future research:

1. Adoption of two-stage ditches could lend itself to a situation of a group of landowners in a watershed making joint decision about adopting/rejecting them. Thus, as much as it fits the “optional” type of innovation-decision identified in the diffusion of innovations literature, it could also fit the “collective” type of innovation-decision. With this in mind, scholars could also test the applicability of the theory of collective action in understanding adoption of two-stage ditches.
2. Scholars should consider testing the perceived attributes of observability and trialability in affecting the adoption of two-stage ditches.

As scholars continue to investigate how to encourage adoption of innovations, it will continue to be important to examine change agents’ perceptions of attributes of the innovation. This is especially important as we see billions of dollars invested in recent decades for promoting and incentivizing the adoption of conservation practices [10].
